# Crosslinking effect of borax additive on the thermal properties of polymer-based 1D and 2D nanocomposites used as thermal interface materials

**DOI:** 10.1038/s41598-022-19755-8

**Published:** 2022-09-26

**Authors:** Geyang Chen, A. A. Yadav, In-Woo Jung, Junho Lee, Kyungwho Choi, Seok-Won Kang

**Affiliations:** 1 Department of Mechanical Engineering, Yeungnam University, Gyeongsan, Gyeongbuk 38541 Republic of Korea; 2 Department of Automotive Engineering, Yeungnam University, Gyeongsan, Gyeongbuk 38541 Republic of Korea; 3 School of Aerospace and Mechanical Engineering, Korea Aerospace University, Goyang, Gyeonggi-do 10540 Republic of Korea

**Keywords:** Mechanical engineering, Polymers, Surfaces, interfaces and thin films, Carbon nanotubes and fullerenes, Synthesis and processing

## Abstract

Recently, polymer-based materials have been used in various filed of applications, but their low thermal conductivity restricts their uses due to the high interfacial thermal resistance. Therefore, in this study, one-dimensional thin-walled carbon nanotube (1D-TWCNT) and two-dimensional boron nitride nanosheet (2D-BNNS) fillers were used to enhance the thermal properties of polyvinyl alcohol (PVA). An important factor to be considered in enhancing the thermal properties of PVA is the interfacial configuration strategy, which provides sufficient pathways for phonon transport and the controlled loss of the intrinsic thermal properties of the filler nanomaterial. In this study, the effect of sodium tetraborate (borax) additive on the thermal properties of 1D-TWCNT/PVA and 2D-BNNS/PVA nanocomposites was explored. Borax is a well-known crosslinking additive that can be used with PVA. The crosslink density of the PVA-borax nanocomposite was controlled by changing its borate ion concentration. The addition of borax into nanocomposites improves the conductivity of 1D-TWCNT/PVA nanocomposites up to 14.5% (4 wt.% borax) and of 2D-BNNS/PVA nanocomposite up to 30.6% for BNNS (2 wt.% borax). Thus, when borax was added, the 2D-BNNS/PVA nanocomposite showed better results than the 1D-TWCNT/PVA nanocomposite.

## Introduction

Thermal management has become increasingly important because of the excessive heat dissipation resulting from the enhanced performance requirements and high-density integration of electrical/electronic devices^1^. The gaps or voids are present between the electronic component and heat sink because of the non-uniform surface, which results in the high thermal interface (Kapitza) resistance^2^. Therefore, the thermal interface material (TIM) used between the heat source and heat sink plays an important role in effectively dissipating heat^3,4^. Polymer-based materials are commonly used in TIM, especially because of their ease of processing, flexibility, and low cost. However, the thermal conductivity of polymer-based materials is low (e.g., 0.19 W/m∙K). Therefore, the incorporation of highly thermally conductive filler nanomaterials such as graphene, carbon nanotubes (CNTs), boron nitride, etc. into the polymer (matrix) to use as TIM is one of the current research trends to improve the contact area between the electronic component and heat sink^1^. In this study, one-dimensional thin-walled CNT (1D-TWCNT) and boron nitride (BN) were used as filler materials.

One-dimensional CNTs show excellent characteristics in improving thermal conductivity because of their superior thermal conductivity of 1000–3000 W/m∙K^5,6^ in nature. Thermal conductivity is strongly dependent on the formation of a continuous conductive network inside the material. However, an increase in the content of CNTs causes interfacial phonon scattering resulting from junctions of CNTs and base materials (e.g., polymers), which can limit the increase in thermal conductivity. Therefore, it is necessary to control the fraction of the filler to sufficiently maintain the mechanical and other properties of the composite while maintaining a high thermal conductivity. In various literatures, CNT-based nanocomposites with various polymer matrices such as polyvinyl alcohol (PVA), polydimethylsiloxane (PDMS), polyimide (PI), polystyrene (PS), polycarbonate (PC), and epoxy were proposed as TIM materials^7–12^. In addition, although CNTs have many advantages for heat conduction, their use in industrial applications requiring electrical insulation of thermally conductive materials is limited, especially because of their metallic or semiconducting nature.

Two-dimensional BN is considered a promising candidate as a filler because they are excellent electrical insulators with high chemical inertness and interlayer interactions, along with excellent thermal and mechanical properties similar to those of CNTs^13,14^. Thus, it can be used as a filler to enhance the low thermal conductivity of polymers in TIM^15–19^. h-BN (hexagonal BN) has a thermal conductivity as high as 400 W/m∙K at room temperature^20^, which is higher than those of most metal and ceramic materials. h-BN has typical anisotropy characteristics in thermo-physical properties (i.e., thermal conductivity): a high in-plane thermal conductivity of 300–600 W/m∙K in the direction parallel to the crystal plane and a relatively low through-plane thermal conductivity of 20–30 W/m∙K in the direction perpendicular to the crystal plane. A BN nanosheet (BNNS) has a two-dimensional (2D) structure, similar to the geometric structure of graphene^21^. These 2D structures can be stacked and held together through van der Waals forces, resulting in several layers of boron nitride nanosheets. Therefore, it is important to arrange each layer to effectively form a thermal transport network between the heat sources and sinks in the use of BN nanosheets for TIM applications. The enhancement of the thermal properties of PVA by using vertically/horizontally aligned BNNS with anisotropic characteristics has been investigated in previous studies^15,16^. In this study, a 2D-BNNS was used as the filler to obtain a polymer-based nanocomposite. Crosslinking of PVA using a crosslinker, such as borax, can also be used to enhance the thermal properties of PVA. Crosslinking of PVA using borax is mentioned in the literature^22,23^.

In this study, 1D-TWCNT and 2D-BNNS were used as fillers in a PVA-based polymer matrix to explore the thermal energy transport in nanocomposites by controlling experimental conditions, such as morphological characteristics and additive concentrations, for the reinforced crosslinking of polymer matrix nanocomposites. The effect of the concentration of borax, the cross-linking additive, on the thermal properties of 1D-TWCNT/PVA and 2D-BNNS/PVA was studied. Initially, different types of 1D-TWCNT (TWCNT-1, TWCNT-2, and TWCNT-3) with a PVA matrix were used to evaluate the thermal properties of PVA. Furthermore, the effects of the borax concentration on the 1D-TWCNT/PVA and 2D-BNNS/PVA nanocomposites were studied, and the results were compared.

## Materials and methods

### Materials

Thin-walled carbon nanotubes (TWCNT) (JEIO), boron nitride (h-BN, 99.8%, 5 µm) (US Research Nanomaterials, Inc., Houston, USA), Sodium dodecyl benzene sulfonate (SDBS) (DAEJUNG) Poly(vinyl alcohol) (PVA, Sigma-Aldrich, USA), Isopropyl alcohol (IPA 99.5%, DAEJUNG, Korea), and sodium tetraborate (Borax) (DUKSAN). All chemicals were used without further purification. All experiments were performed using deionized water (DI).

### Synthesis of polymer-based nanocomposites

#### Synthesis of TWCNT/PVA and borax: TWCNT/PVA nanocomposite

**Figure 1:**
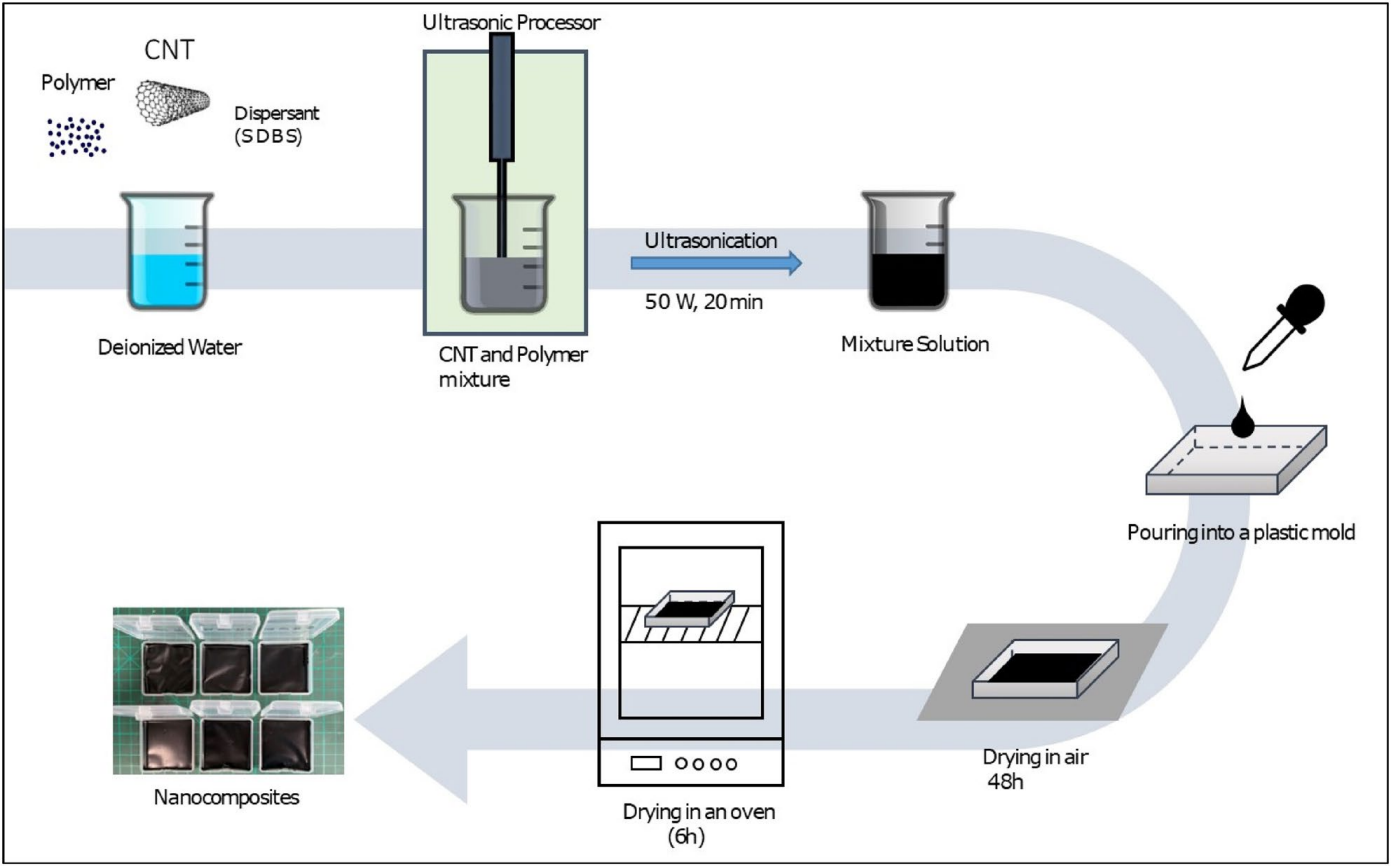
Overall experimental procedure for synthesis of TWCNT/PVA nanocomposite films.

Three types of TWCNTs were used as fillers for the synthesis of TWCNT/PVA nanocomposites: TWCNT-1, TWCNT-2, and TWCNT-3 (Table 1). In particular, PVA was used as the polymer matrix for TWCNT fillers. Initially, 0.2 g of PVA was dissolved into 50 ml of DI with continuous stirring to form a PVA solution. Furthermore, (0.10 g) 0.20 wt.% TWCNTs were dispersed in PVA solution along with SDBS that was used as a dispersant (0.2 g). The TWNT:SDBS:PVA ratio was 1:2:2. A homogeneous reaction mixture was prepared using an ultrasonic probe sonicator at 50 W for 20 min. Next, this reaction mixture was poured into a 25 cm^2^ square plastic mold and dried in air at room temperature for 48 h. To remove residual water, the final TWCNT/PVA was dried in an oven at 40 °C for an additional 6 h, as shown in Fig. 1. Similarly, borax: TWCNT/PVA nanocomposites with different borax weight percentages, such as 2 wt.%, 4 wt.%, and 6 wt.%, were prepared. In a homogenous solution mixture of TWCNT, SDBS, and PVA, 2 wt.%, 4 wt.%, and 6 wt.% borax was added, respectively, and then mixed well using the sonication. The sample was prepared in a similar way as TWCNT:SDBS:PVA. Table 2 shows the experimental parameters for the synthesis of the borax: TWCNT/PVA nanocomposite.

**Table 1:** Specifications of CNTs used for fabrication of nanocomposites.

CNT	Manufacturer	Diameter (nm)	Length (µm)
TWCNT-1	JEIO	5–7	50–150
TWCNT-2		5–20	100–200
TWCNT-3		7–12	100–200

**Table 2:** Concentrations of TWCNT, PVA, SDBS, and borax in nanocomposites.

Fillers	PVA (g)	Fillers (wt.%)	Fillers solid (g)	SDBS (g)	Borax (wt.%)	Borax (g)
TWCNT-1	0.2	20	0.1	0.2	2	0.004
	0.2	20	0.1	0.2	4	0.008
	0.2	20	0.1	0.2	6	0.012
TWCNT-2	0.2	20	0.1	0.2	2	0.004
	0.2	20	0.1	0.2	4	0.008
	0.2	20	0.1	0.2	6	0.012
TWCNT-3	0.2	20	0.1	0.2	2	0.004
	0.2	20	0.1	0.2	4	0.008
	0.2	20	0.1	0.2	6	0.012

#### Synthesis of BNNS/PVA and borax: BNNS/PVA nanocomposite

Using a similar method, BNNS/PVA and borax: BNNS/PVA samples were prepared to study the effect of filler morphology on thermal conductivity. For the synthesis of the BNNS/PVA nanocomposite, 20 wt.% (0.15 g) BNNS was dissolved in the PVA (0.6 g) matrix solution. Next, the stable solution mixture was achieved by homogenization for 20-min using a probe sonicator (500 W, 20 kHz). To prepare the borax: BNNS/PVA nanocomposite, 2 wt.%, 4 wt.%, and 6 wt.% of borax was added, respectively, in the homogenous solution of 20 wt.% of BNNS and PVA. The experimental parameters are listed in Table 3.

**Table 3:** Concentrations of BNNS, PVA, and borax in nanocomposites.

Fillers	PVA (g)	Fillers (wt.%)	Fillers solid (g)	Borax (wt.%)	Borax (g)
BNNS	0.6	20	0.15	2	0.004
	0.6	20	0.15	4	0.008
	0.6	20	0.15	6	0.012

### Material characterization

The sample’s thickness was measured using a thickness gauge (Minutolo, 547-401). The surface morphologies of the prepared samples were studied using field-emission scanning electron microscopy (FESEM, Hitachi S-4800).

### Experimental apparatus and method for evaluation of thermal performance

**Figure 2:**
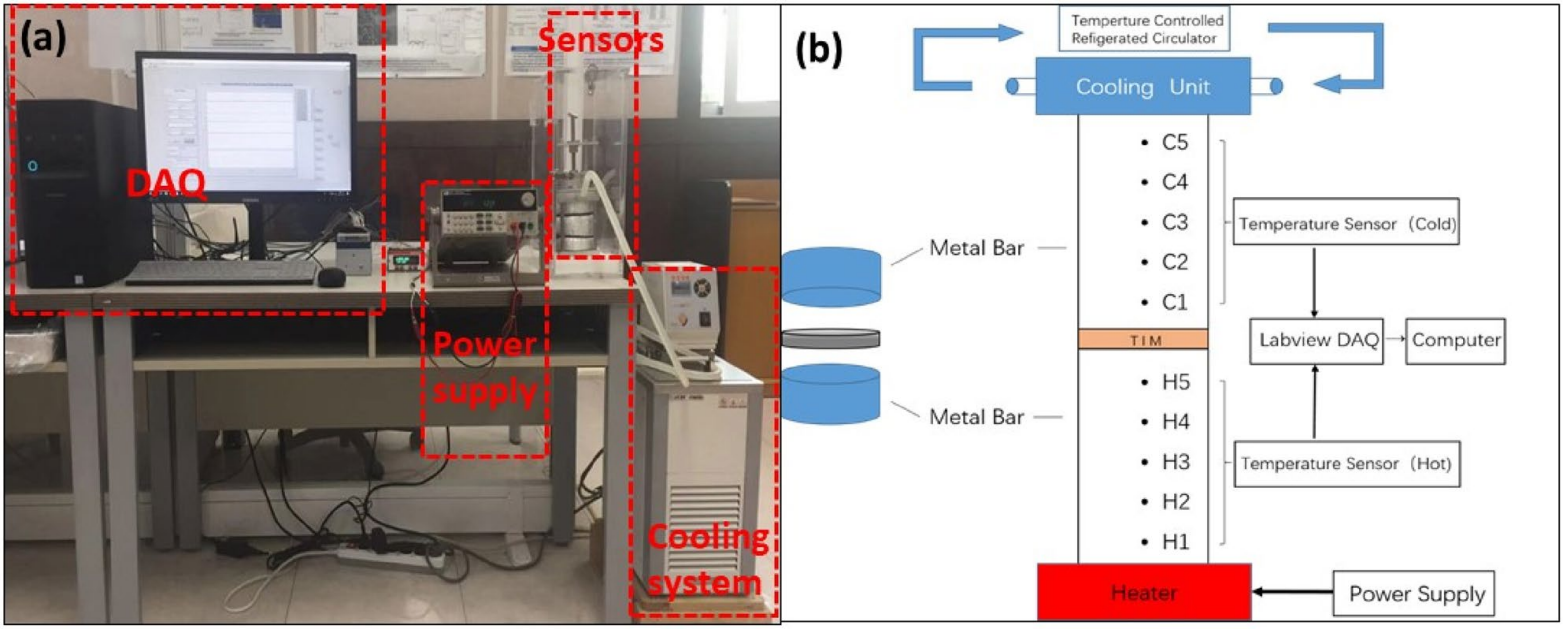
(**a**) Test system for thermal conductivity and (**b**) general features of apparatus used in this method.

The thermal conductivity of the prepared sample was measured using a homemade ASTM D5470 standard setup, as shown in Fig. 2a. Grease or paste materials were loaded onto both sides of the film sample, and then loaded onto the lower test stack. Subsequently, the lead screw compacted the film sample. The heater and cooling units were turned on, and then stabilized at the specified setting so that the average temperature of the test sample was 50 °C (average of T_C1_ and T_H5_), as shown in Fig. 2b. Temperature changes were recorded and stored using temperature sensors and simulation software. At constant power, the average sample temperature was kept steady at 50 ± 2 °C for 30 min, and then thermal conductivity was calculated. The thermal impedance was measured for every sample thrice to minimize the error.

The heat flow through the test film sample was computed as the average heat flow through both the meter bars. The general features of the meter bars are shown in Fig. 2b. The heat flow is calculated as follows:

1 $$Q_{C} = \frac{\lambda \times A}{d} \times \left[ {T_{C1} - T_{C2} } \right]$$

2 $$Q_{H} = \frac{\lambda \times A}{d} \times \left[ {T_{H4} - T_{H5} } \right]$$

3 $$Q = \frac{{Q_{C} + Q_{H} }}{2}$$

where Q_C_ is the heat flow in the cold meter bar, Q_H_ is the heat flow in the hot meter bar, Q is the average heat flow through the film sample, λ is the thermal conductivity of the meter bar and material, A is the area of the reference calorimeter, d is the distance between the temperature sensors (C1 and C2, H4, and H5), T_H4_ is the temperature of sensor H4, T_H5_ is the temperature of sensor H5, T_C1_ is the temperature of sensor C1, and T_C2_ is the temperature of sensor C2.

The temperatures of the hot and cold meter bar surfaces in contact with the film samples were measured using Eqs. (4) and (5), respectively:

4 $$T_{C} = T_{C1} - \frac{d}{{d_{C} }}\left[ {T_{C1} - T_{C2} } \right]$$

5 $$T_{H} = T_{H5} - \frac{d}{{d_{H} }}\left[ {T_{H4} - T_{H5} } \right]$$

where T_C_ is the temperature of the film sample surface in contact with the cold meter bar, T_H_ is the temperature of the film sample surface in contact with the hot meter bar, d_C_ is the distance from T_C1_ to the film sample surface in contact with the cold meter bar, d_H_ is the distance from T_H5_ to the film sample surface in contact with the hot meter bar, and L is the thickness of the film sample. The thermal conductivity was calculated using Eq. (6).

6 $$k = \frac{Q \times L}{{A \times \left[ {T_{H} - T_{C} } \right]}}$$

The thermal conductivity was expressed in units of W/m∙K.

## Results and discussion

**Figure 3:**
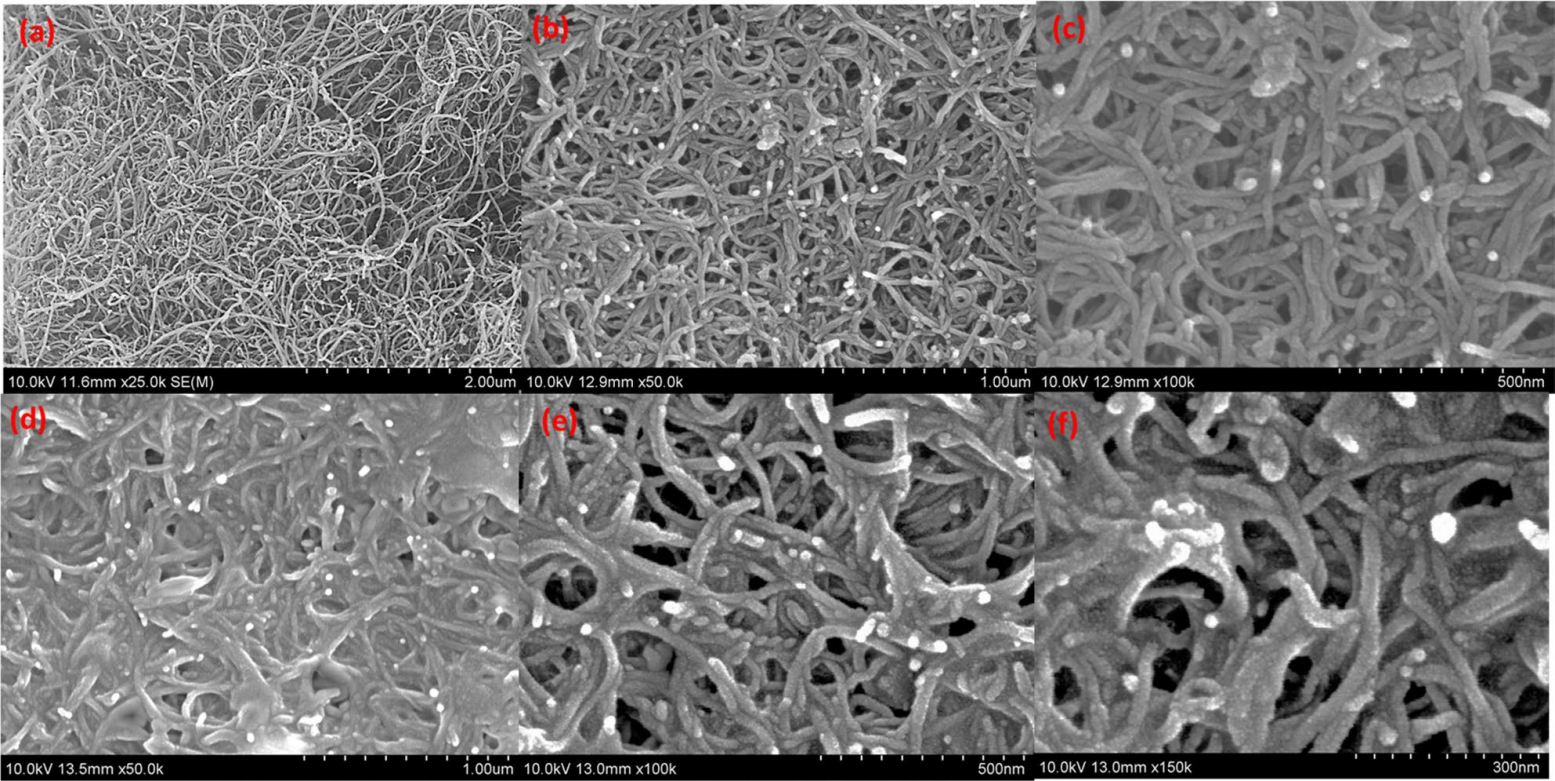
FE-SEM images for (**a**) TWCNT, (**b**,**c**) TWCNT/PVA, and (**d**–**f**) borax: TWCNT/PVA.

TWCNT-filled polymer matrix nanocomposites have demonstrated an anomalous thermal conductivity enhancement beyond the expected values based on the concentration of CNTs^24^. This is because of the unique structure of CNTs with a high aspect ratio, and the conductivity mainly attributed to the low-frequency phonons, depending on their high phonon velocities and the large mean free paths. When these CNTs are assembled, their interfacial thermal resistance adversely affects thermal energy transport. According to the literature, when CNTs are assembled in the form of a bundle, the conductivity of the assembly is only one-third of the conductivity of an individual CNT as shown in Fig. 3a. When CNTs are assembled perpendicularly, the conductivity of the assembly is smaller than that of an individual CNT by two orders of magnitude. The interfacial thermal resistance between two CNTs depends on the type of contact between them, that is, whether they are parallel or perpendicular to each other. In addition, when the CNT forms a composite with PVA, the thermal conductivity of the composite decreases by approximately 30–50%, indicating that the heat transfer among the CNT bundles is inhibited by the polymer^25^. The morphological images of the TWCNT/PVA nanocomposite are shown in Fig. 3b,c To enhance the thermal properties of PVA, it was crosslinked using borax and the FE-SEM images of borax: TWCNT/PVA are shown in Fig. 3d. The magnified images of borax: TWCNT/PVA (Fig. 3e,f) show improved cross-linking of the PVA matrix after the addition of borax. The morphological study shows the change in the size of latex particles of PVA after addition of borate ions, similar effect observed previously for poly (vinyl acetate) matrix^23^. By adding borax to an aqueous solution of PVA, the gelation of PVA can be initiated, which can be attributed to the formation of a network among the borate ions and (–OH) in the adjacent polymer strands. The crosslinking reaction takes place expressed as follows^22^:7

**Figure 4:**
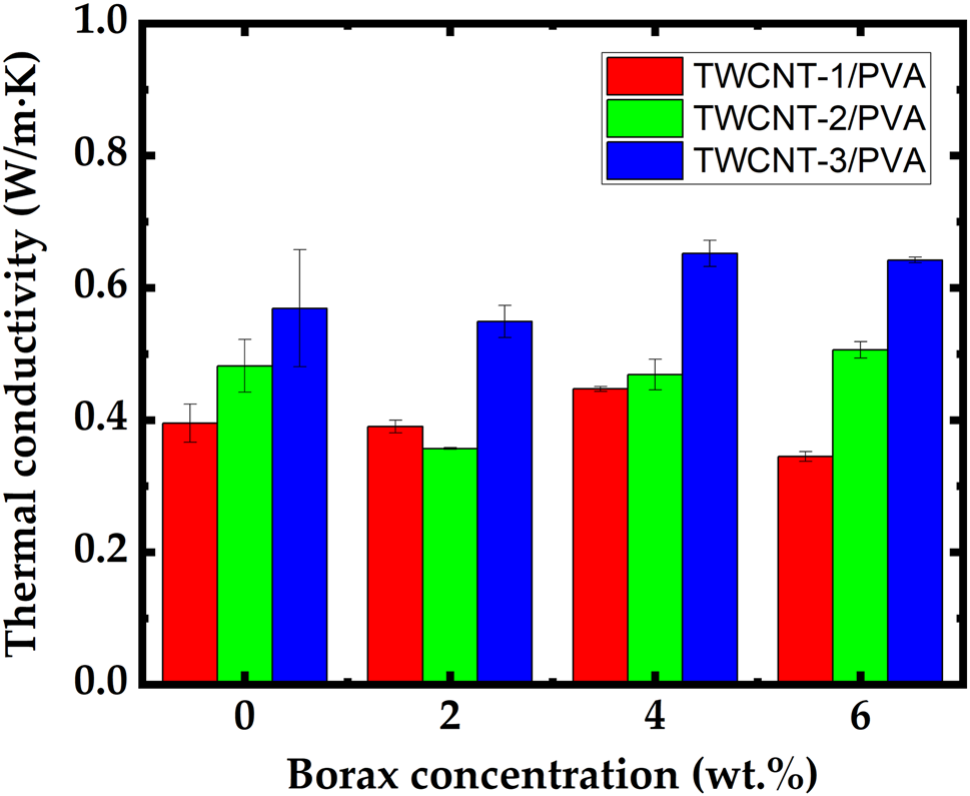
Comparison of thermal conductivities of TWCNT/PVA for (TWCNT-1, TWCNT-2, and TWCNT-3) at various concentrations of borax (0, 2, 4, and 6 wt.%).

The crosslinking density of the borax–PVA composite can be adjusted by changing its borate ion concentration. In this study, the thermal properties of three different TWCNTs/PVA (TWCNT-1, TWCNT-2, and TWCNT-3) were studied. To reduce the production cost and agglomeration caused by high filler concentrations, which affects the flexibility and processability of nanocomposite films, the TWCNT filler concentration was set as 20 wt.% based on the results of a previous investigation^7^. As shown in Fig. 4, the TWCNT/PVA (TWCNT-3) composite exhibited the highest thermal conductivity (0.569 W/m∙K). The crosslinking density of the composite was varied by changing the borax concentration to 2, 4, and 6 wt.%. It has been known that the crosslinking effect can cause two separate impacts on the PVA molecular structures: (i) immobilization of chain segments of PVA at the chelate points between the OH groups and the borate ions and (ii) a copolymer effect resulting in depression of crystallinity^22^. The thermal conductivity was calculated for the different nanocomposites of TWCNT/PVA at various concentrations of borax as shown in Fig. 4.

**Figure 5:**
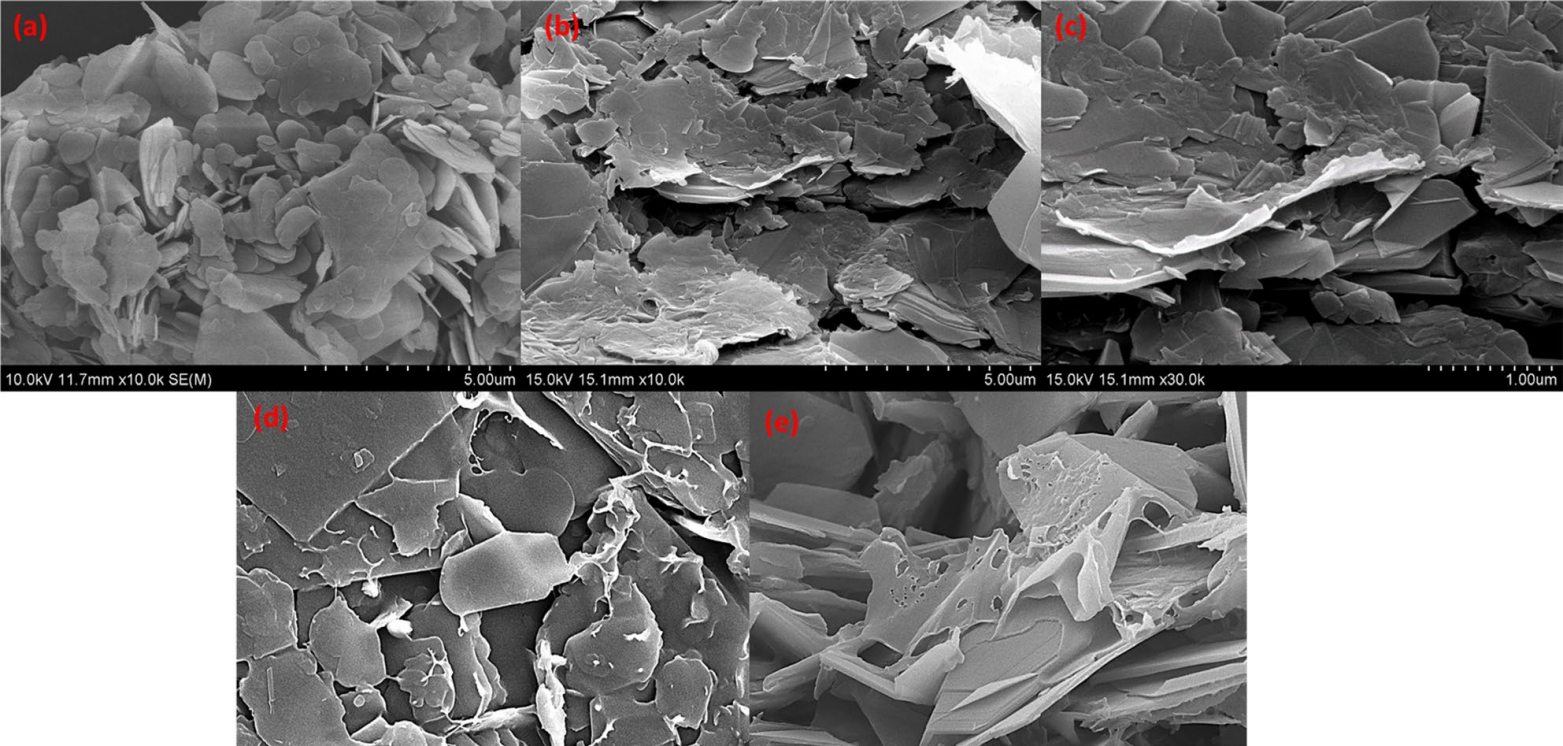
FE-SEM images of (**a**) BN, (**b**,**c**) BNNS/PVA, (**d**,**e**) borax: BNNS/PVA.

TWCNT/PVA nanocomposite films added by borax of 4 and 2 wt.% were shown in Fig. 4, respectively. As a result, the thermal conductivity was improved by 14.5% (0.652 W/m∙K), compared to the bare TWCNT/PVA nanocomposite. In particular, enhancement was demonstrated for all TWCNT geometry types. Thus, adding borax to the PVA matrix enhanced its thermal conductivity, and the maximum thermal conductivity of the borax: TWCNT/PVA nanocomposite obtained using a 4 wt.% borax concentration with the TWCNT-3 sample was 0.652 W/m∙K. Thermal properties of borax: TWCNT/PVA nanocomposite was further compared with the PVA matrix with 2D filler BNNS at different concentrations of borax. Figure 5a shows the morphological characteristics of BN. The FE-SEM images for the bare BNNS/PVA were shown in Fig. 5b,c. To improve the crosslinking of PVA matrix borax additive with different concentrations such as 2, 4, and 6 wt.% was added. The enhanced crosslinking is easily observed in Fig. 5d,e. The thermal conductivity of borax: BNNS/PVA nanocomposite was calculated and compared with borax: TWCNT/PVA (TWCNT-3) nanocomposite.

**Figure 6:**
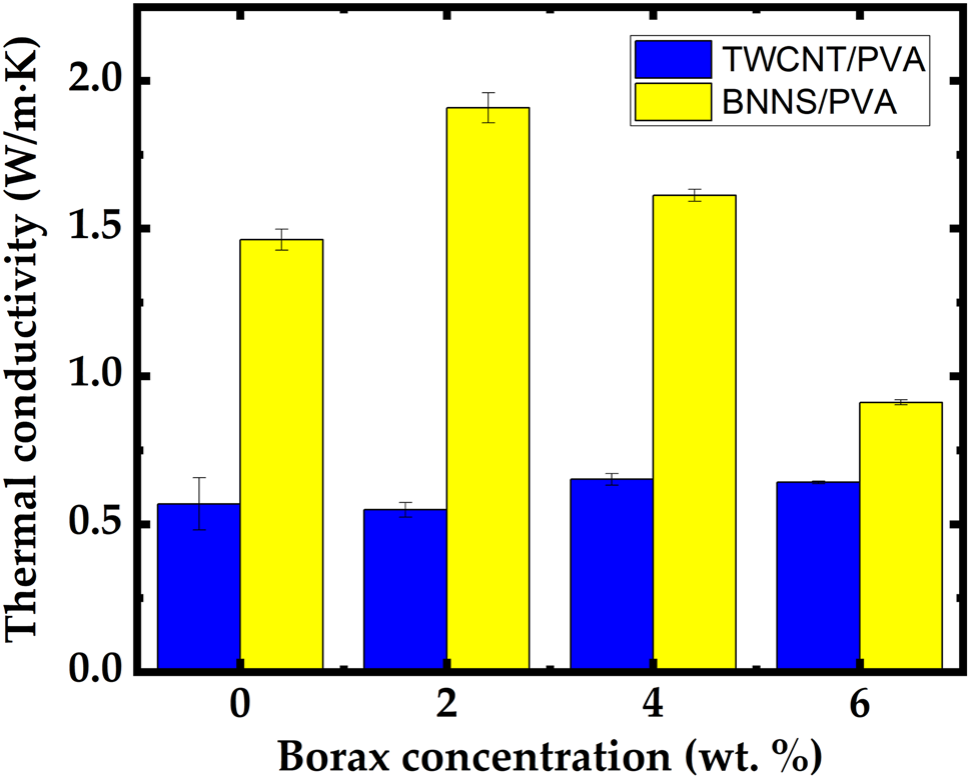
Comparison of the thermal conductivities of TWCNT/PVA and BNNS/PVA nanocomposite films obtained using different borax concentrations (2–6 wt.%).

From the measurement results shown in Fig. 6, it was confirmed that the thermal conductivity for pure BNNS/PVA was 1.4169 W/m∙K (± 0.036). The thermal conductivity enhanced for the borax: BNNS/PVA nanocomposite, the maximum thermal conductivity obtained at the 2 wt.% is 1.909 W/m∙K (± 0.007). In particular, the excellent performance of BNNS at the same concentration (20 wt.%) can be analyzed by comparing the microstructures of two nanocomposite films. First, the cylindrical TWCNTs with a high aspect ratio were easily tangled when they were dispersed in a PVA/DI (de-ionized water) mixture solution, compared to BNNS with a flake shape. It was found that the increase in junction points between TWCNTs resulted in phonon scattering at their interfaces, even if the thermal conductivity of a single TWCNT nanoparticle was much higher than that of a single BNNS nanoparticle. In addition, because TWCNTs are hydrophobic in water, a dispersant (e.g., SDBS: Sodium Dodecyl Benzene Sulfonate) was used to enhance colloidal stability. The presence of dispersants inside the composite films negatively affected the thermal conductivity. Unlike TWCNTs, nanostructured BN has hydrophilic characteristics and a good affinity for DI. Furthermore, BNNS is a 2D crystalline form with strong bonds and weak van der Waals forces between the layers^26^. Thus, flake-shaped BNNS can form a stacked structure with fewer voids (Fig. 5d) than the entangled structure between the cylindrical TWCNTs (Fig. 3d). Air voids can cause more disconnections between the thermally conductive pathways because voids act as barriers to phonon transport. The PVA-based nanocomposites using TWCNT and BNNS as fillers, as summarized in Table 4, were improved by approximately 285% (0.569 W/m∙K) and approximately 730% (1.462 W/m∙K), respectively, compared to pure PVA (0.2 W/m∙K).

**Table 4:** Results of thermal conductivity measurements of nanofiller/PVA composites and comparison of data from previous studies.

Polymers	Fillers (wt.%)	Thermal conductivity (W/m∙K)	Method of measurement	Note	References
PVA	TWCNT (20)	0.652	ASTM D5470	–	Current study
	TWCNT (15)	~ 0.2	ASTM D5470	–	^7^
	TWCNT (30)	~ 0.4			
PDMS	MWCNT (2)	0.2748	MTPS	–	^8^
PI	SWCNT (5)	0.334	THW	–	^9^
PS	MWCNT (1)	0.1847	THW	–	^10^
	MWCNT (2)	0.1891			
	MWCNT (3)	0.1911			
PC	Long MWCNT (2)	1.27	MTPS	Aligned	^11^
Epoxy	SWCNT (1)	0.49	ASTM D5470	–	^12^
PVA	BN (20)	1.91	ASTM D5470	–	Current Study
	h-BN (30)	4.41	LFA	Aligned	^15^
	h-BN (10)	5.4	LFA	Aligned	^16^
Epoxy	h-BN (30)	0.6	LFA	–	^17^
	h-BN (50)	0.478	LFA	–	^18^
	BN (50)	1.52	ASTM D5470	–	
PCL	h-BN (20)	1.96	LFA	–	^19^

It is known that borax contributes to the creation of a cross-linked bonding network between the PVA molecular chains and enhancement of the viscoelasticity of PVA^27,28^. In addition, because hydrogels formed by the PVA-borax complexation have high inherent water solubility, it seems that the dispersibility of filler nanoparticles in the solution mixture was maintained despite the increase in its viscoelastic characteristics^22^. It was confirmed that when 4 wt.% and 2 wt.% were added to the TWCNT/ PVA (TWCNT-3) and PVA/BNNS nanocomposite films, respectively, the thermal conductivity was improved by 14.5% (0.652 W/m∙K) and 30.6% (1.909 W/m∙K), respectively, compared to before the addition of borax.

**Figure 7:**
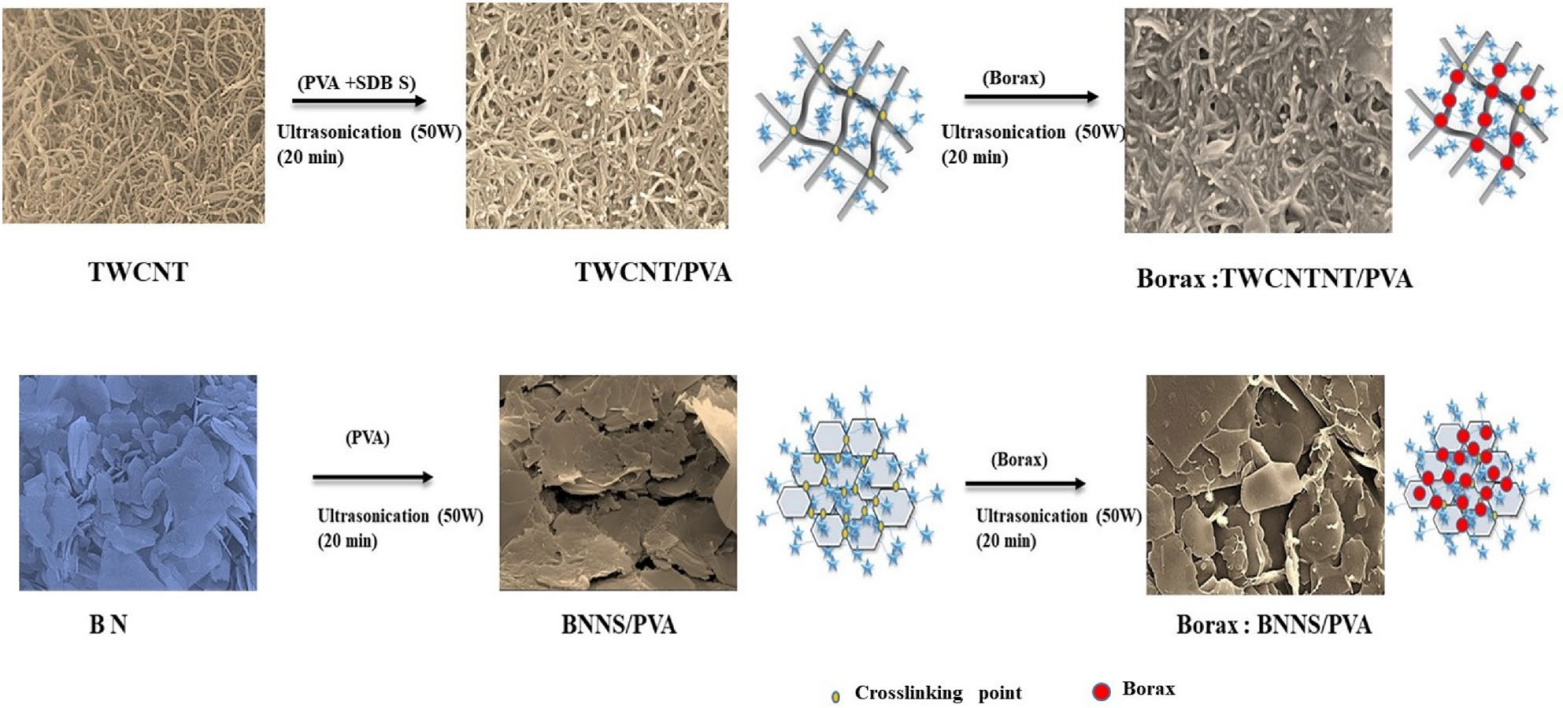
Enhancement mechanism through reinforced crosslinking and formation of thermal transport network by adding borax.

Figure 7 shows the schematic of the mechanism of thermal conductivity enhancement after the addition of borax. This is because borax strengthens the cross-linking between the fillers and promotes the formation of the heat conduction network. The addition of borax can cost-effectively reduce the directional randomness of the fillers in the nanocomposites. However, it has a limitation in that it cannot produce an optimized value for the thermal conductivity of the nanocomposites^29,30^. Even if alignment considerably improves their thermal conductivity (for example, ~ 1.27 W/m∙K^11^), it is costly and unsuitable for large-scale production. The approach proposed in this study has commercial advantages because of the simple and inexpensive synthesis protocol.

Because the proposed method cannot be used to change the microstructure of the composite directly, the performance of the composite considerably depends on the concentration of the additives (i.e., borax). Accordingly, the enhancement of the thermal conductivity was limited for nanocomposite films when the concentration of additives was above the optimal value (i.e., 4 wt.% for TWCNT and 2 wt.% for BNNS), as shown in Figs. 4 and 6. This is because excessive borax causes an increase in the viscosity of the PVA/borax mixture solution, which results in a negative effect on the homogeneous dispersion of filler nanoparticles. Therefore, it can be concluded that there is an appropriate concentration of borax additive according to the shape of the filler nanoparticles.

## Summary and conclusion

In this study, an experimental investigation of the thermal performance of PVA matrix nanocomposite films was successfully conducted using two different fillers, 1D-TWCNT and 2D-BNNS, to determine the TIM applications of the nanocomposite films. A study on the thermal conductivity of three different types of TWCNT/PVA nanocomposites (TWCNT-1, TWCNT-2, and TWCNT-3) was conducted. The effect of borax, added as a crosslinker at different concentrations (2, 4, and 6 wt.%), on the thermal conductivity of the TWCNT/PVA nanocomposite was studied. The results showed that the thermal conductivity of the nanocomposite (TWCNT-3) improved at a borax concentration of 4 wt.%. Similarly, the thermal properties of the BNNS/PVA nanocomposites were studied using 2, 4, and 6 wt.% borax concentrations. The maximum thermal conductivity of the nanocomposites was obtained at a borax concentration of 2 wt.%. The degree of crosslinking in the PVA matrix is correlated with borax concentration. The PVA latex particle size and plasticizing effect are crucial factors that contribute to the high thermal conductivity of the TWCNT/PVA and BNNS/PVA nanocomposites at borax concentrations of 4 and 2 wt.%, respectively. Borate functions as a good crosslinker in the polymer matrix, and thus the impact of its addition to the polymer matrix can be neglected. The thermal conductivity of the nanocomposite films using flake-shape BNNS fillers was found to be superior (i.e., 7.31 times the thermal conductivity of PVA) to that of nanocomposites using the cylindrical TWCNT fillers at the same concentrations (20 wt.%). Further increase in the borax concentration reduced the thermal conductivity of the nanocomposites. The study findings show that the Borax-PVA matrix with a 2D BNNS filler would be a potential candidate for TIM applications.

## Data Availability

The datasets used and/or analyzed during the current study are available from the corresponding author on reasonable request.
